# Behavior of Osteoblastic Lineage Cells When in the Presence of Tamoxifen: In Vitro and In Vivo Studies on Osseointegration

**DOI:** 10.3390/dj13080351

**Published:** 2025-08-01

**Authors:** Luiz Guilherme Fiorin, Emanuela Galliera, Henrique R. Matheus, Dolaji Henin, Edilson Ervolino, Gabriela Carrara Simionato, Juliano Milanezi de Almeida, Claudia Dellavia

**Affiliations:** 1 Department of Diagnosis and Surgery, Division of Periodontics, São Paulo State University “Júlio de Mesquita Filho”—UNESP, Araçatuba 16015-050, Brazil; guilherme@fior.in (L.G.F.); hrmatheus@hotmail.com (H.R.M.); gabriela.carrara@unesp.br (G.C.S.); 2 Thin Section Laboratory, Department of Biomedical, Surgical and Dental Sciences, Università degli Studi di Milano Statale (UNIMI), 7-20122 Milan, Italy; dolaji.henin@unimi.it (D.H.); claudia.dellavia@unimi.it (C.D.); 3 Department of Biomedical Sciences for Health, Università degli Studi di Milano, 7-20122 Milan, Italy; emanuela.galliera@unimi.it; 4 Istituto di Ricovero e Cura a Carattere Scientifico (IRCCS)—Istituto Ortopedico Galeazzi, 20157 Milan, Italy; 5 Discipline of Periodontics, School of Dentistry, University of São Paulo, São Paulo 05508-000, Brazil; 6 Department of Basic Science, São Paulo State University (UNESP), School of Dentistry, Araçatuba 16018-805, Brazil; e.ervolino@unesp.br

**Keywords:** tamoxifen, selective estrogen receptor modulators, cell culture, titanium implants

## Abstract

**Background/Objectives:** Tamoxifen, a selective estrogen receptor modulator widely used as an adjunct in the treatment of breast cancer, has known effects on bone metabolism, although its impact on osseointegration and cellular responses during early bone healing remains unclear. Understanding these effects is essential given the increasing use of dental implants in cancer survivors. The study aimed to observe the influence of tamoxifen on human osteosarcoma (SAOS-2) cells lines, as well on the osseointegration of titanium implants in ovariectomized female rats. **Methods:** SAOS-2 cells were incubated with Dulbecco’s modified growth medium. Six titanium (Ti) disks were used at each time point. The samples were divided into groups with the presence (TAM, *n* = 36) or not (CTR, *n* = 36) of tamoxifen in a concentration of 2 μM. In vivo, 72 animals were divided in groups with bilateral ovariectomy or SHAM and tamoxifen administration or not (15 mg/kg). Cell viability, mineralization rate, and collagen synthesis were assessed, as well as bone/implant contact (BIC) and bone ingrowth (BIN). **Results:** Tamoxifen caused a decrease in SAOS-2 viability, although an increase in the mineralization rate was observed. In vivo, the TAM groups presented higher BIC and BIN when compared to their control, but a lower percentage of mature collagen cells. **Conclusions:** Based on our findings, in vitro, the therapy with TAM slightly reduced the viability of SAOS-2 cells while significantly increasing the mineralization rate. In vivo, the therapy positively influenced BIC and BIN during the osseointegration phase.

## 1. Introduction

Originally investigated as a contraceptive agent, tamoxifen was repurposed in the late 1970s and became the first selective estrogen receptor modulator (SERM) [1], as well as one of the earliest targeted therapies for breast cancer. Due to its well-established efficacy in the treatment of estrogen receptor-positive (ER+) tumors and a more favorable side-effect profile compared to other SERMs [2,3,4], tamoxifen remains widely used as an adjuvant therapy [5,6].

Estrogen receptors, which are expressed in various tissues [7], are classified into two subtypes—ERα and ERβ—and function primarily by regulating gene transcription. Although antiestrogenic agents typically compete with endogenous estrogens to block receptor activation and exert antagonistic effects, prior studies have demonstrated that tamoxifen exhibits tissue-specific activity, acting as either an agonist or antagonist depending on the target tissue [8,9,10]. Moreover, tamoxifen not only activates both estrogen receptor subtypes [11], but also exerts its effects through multiple molecular pathways, including the inhibition of protein kinases and the activation of apoptotic signaling cascades.

The effects of tamoxifen on bone tissue are multifactorial and appear to be influenced by both hormonal status and treatment duration, which can vary between 5 and 10 years [12,13]. In postmenopausal women, it tends to act as an estrogen agonist in bone, reducing bone turnover and preserving bone mineral density [14,15,16,17]. On the other hand, in premenopausal women, tamoxifen may act more as an antagonist, leading to bone loss during treatment [18,19]. Although these effects are well described in relation to systemic bone health, little is known about how tamoxifen might influence localized bone repair processes, such as those involved in implant osseointegration.

Bone metabolism plays a fundamental role in the success of dental implants. As introduced by Branemark [20], the concept of osseointegration refers to the direct structural and functional connection between living bone and the surface of a titanium dental implant. The long-term stability of implants depends not only on this intimate contact but also on the bone’s ability to adapt and remodel in response to functional load [21]. Although tamoxifen reportedly stimulates osteoblast activity and its mineralization [22,23], maintains bone mass, and positively influences the remodeling process of titanium implants [24], there is limited evidence on the influence of tamoxifen in early bone healing and osseointegration.

The aims of the present study were (i) to evaluate in vitro the response of osteoblasts to tamoxifen in terms of viability, collagen synthesis, and mineralizing capacity; and (ii) to assess in vivo the effect of systemic tamoxifen administration on titanium implant osseointegration.

## 2. Methods

### 2.1. In Vitro Evaluation

#### 2.1.1. Experimental Groups and Experimental Design

Human osteogenic sarcoma cells (SAOS-2 cell line; Sigma-Aldrich, 89050205, Saint Louis, MO, USA) were used for all in vitro experiments [25,26]. Cells were cultured under three specific conditions, according to the intended analysis:

- In complete Dulbecco’s modified Eagle medium (DMEM) supplemented with 10% fetal bovine serum (FBS), 10,000 IU/mL penicillin G sodium, 100,000 μg/mL streptomycin sulfate, 25 μg/mL amphotericin B, and 1% L-glutamine—for cell viability, morphological evaluation, and collagen synthesis analysis.
- In DMEM without FBS—for collagen synthesis analysis.
- In osteogenic medium (OM), consisting of DMEM supplemented with 50 μg/mL ascorbic acid and 10 mM β-glycerophosphate—for mineralization assays.

For subculturing, adherent cells were detached by removing the culture medium, gently washing with sterile saline, and incubating with 0.5 mL of trypsin per 10 cm^2^ at room temperature for approximately 5 min. Cell detachment was confirmed by light microscopy, and the enzymatic activity was neutralized with twice the volume of pre-warmed complete medium.

The cell suspension was then centrifuged at 200× *g* (1000 RPM) for 5 min. The resulting pellet was resuspended in a minimal volume of complete medium, and cell concentration and viability were assessed using a hemocytometer (Neubauer chamber; Sigma-Aldrich, St. Louis, MO, USA). The final suspension was adjusted to a density of 140,000 cells/mL. Approximately 1 mL of this suspension (total volume 17 mL) was added to each well and allowed to stabilize for 48 h (Day 0) under standard incubation conditions (37 °C, 5% CO_2_).

Cells were seeded at a density of 2.4 × 10^4^ per well on the surface of titanium discs (grade 4, Ø 20 mm × 5 mm height), previously treated by CaMg(CO_3_)_2_ blasting, plasma decontamination, and UV sterilization for 20 min on each side. Each disc was placed in the center of a six-well plate. A total of 72 discs were used (6 per time point).

Experimental groups were divided into control (CTR) and tamoxifen-treated (TAM). CTR samples were cultured in OM without tamoxifen, while TAM samples were cultured in OM supplemented with 2 µg/L tamoxifen (Enzo Life Sciences, Long Island, New York, USA) [27]. For viability testing, tamoxifen was administered for up to 96 h. For collagen synthesis and mineralization assays, it was administered for up to 14 days.

**Figure 1:**
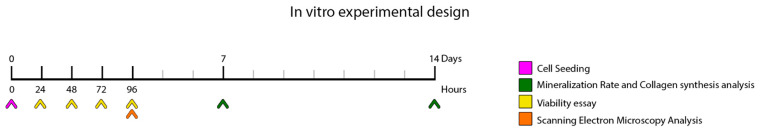
Schematic representation of the in vitro experimental setup, illustrating the workflow and procedures performed during the laboratory phase of the study. At time point 0, cells were seeded. Cell viability was assessed at 24, 48, 72, and 96 h post-seeding. At 96 h, samples were processed for scanning electron microscopy (SEM) analysis. Mineralization rate and collagen synthesis were evaluated at 7 and 14 days following cell seeding.

A timeline is included to illustrate the in vitro experimental design (Figure 1). All experiments were performed in triplicate.

#### 2.1.2. Viability

The viability was assessed at 24, 48, 72 h, and 96 h using Alamar Blue (Life-Technologies, Carlsbad, California, USA). Alamar Blue (100 μL) was added to 900 μL of cells+ medium in a 6 microplate wells (Thermo Fisher Scientific, Waltham, MA, USA) and then returned to the incubator at 37 °C. After 4 h, the supernatant was collected and added to a 96-well microplate, and absorbance was read in triplicate at the wavelengths of 560 nm and 600 nm (GloMax Plate Reader, Madison, WI, USA). The percentage of viable cells was calculated as the difference in the reduction between the test group and a control sample [28,29].

The following formula was used:

$$\%Viability\;=\;\frac{\left(O_{2}\;\times \;A_{1}\right)\;-\;\left(O_{1}\;\times \;A_{2}\right)}{\left(R_{1}\;\times \;N_{2})\;-\;(R_{2}\;\times \;N_{1}\right)}\;\times \;100$$

where

O_1_ = Oxidized Alamar Blue^®^ coefficient at 560 nm = 80.586.
O_2_ = Oxidized Alamar Blue^®^ coefficient at 600 nm.
R_1_ = Reduced Alamar Blue^®^ coefficient at 560 nm.
R_2_ = Reduced Alamar Blue^®^ coefficient at 600 nm.
A_1_ = Absorbance of the sample at 560 nm.
A_2_ = Absorbance of the sample at 600 nm.
N_1_ = Absorbance of the negative control (media + reagent, no cells) at 560 nm.
N_2_ = Absorbance of the negative control at 600 nm.

#### 2.1.3. Morphological Analysis

The morphological analysis was performed at 96 h. The medium was removed from the 6-well microplate, and samples were fixed in a solution of glutaraldehyde 2% and cacodylate 0.1 M for 2 h at 4 °C, soaked in a cacodylate 0.1 M buffer for 2 h, dehydrated in serial ethanol grading and with hexamethyldisilane (HMDS), and finally gold-sputtered. Samples were then analyzed by SEM microscope (Jeol Neoscope Electron Microscope JCM-6000, Tokyo, Japan) to evaluate cell morphology. A qualitative morphological analysis was assessed on images captured at 500× magnification on the secondary electron imaging (SEI) mode for the observation of surface features.

#### 2.1.4. Collagen Synthesis

The cells cultivated in the 6-well microplate were fixed with Bouin solution (Sigma-Aldrich) for 1 h and then stained with 0.1% Sirius Red (F3 BA-Sigma-Aldrich) dissolved in picric acid for 1 h. The stained cells were incubated in 0.1 M NaOH for 10 min. The supernatant was collected, added to a 96-well microplate, and absorbance was read in triplicate at the wavelength of 550 nm (GloMax Plate Reader). The rate of collagen synthesis was calculated by comparing samples to a sodium hydroxide control [30].

#### 2.1.5. Mineralization

Samples were fixed in 70% ethanol at 4 °C for 1 h, followed by washing in PBS and staining with 40 mM Alizarin Red (Sigma-Aldrich) solution for 20 min. Samples underwent five-time washing in deionized water under shaking for 15 min, immersed in 10% cetylpyridinium chloride, and incubated at room temperature for 20 min. The supernatant was collected, added to a 96-well microplate, and absorbance was read in triplicate at the wavelength of 570 nm (GloMax Plate Reader). The mineralization rate was calculated by comparing the samples to a sodium hydroxide control [30].

### 2.2. In Vivo Study Design and Experimental Groups

**Figure 2:**
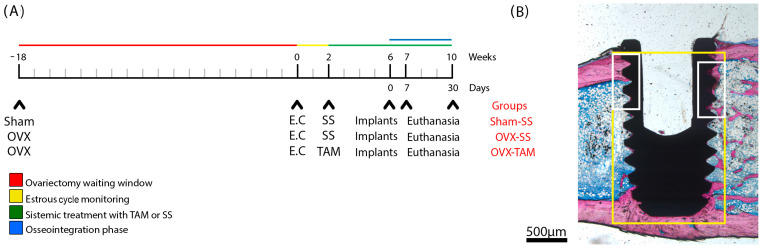
Study design, surgical protocol for implant installation, and delineation of regions of interest (ROIs). (**A**) Timeline of the in vivo experiments. Three study groups were evaluated: SHAM (sham-operated) and OVX (bilateral ovariectomy). Experiments began at week 0, which corresponds to 18 weeks post-ovariectomy. At week 0, estrous cycle monitoring was conducted over a 2-week period. At week 2, the experimental groups received treatment for 8 weeks with either saline solution (SS) or tamoxifen (TAM). Implants were placed at week 6. Animals were euthanized at weeks 7 and 10. The osseointegration phase was defined as the period between weeks 6 and 10. (**B**) Schematic representation of the ground section highlighting the regions of interest: ROI 1 (yellow) and ROI 2 (white).

A schematic timeline (Figure 2) illustrates the experimental design, treatment schedule, and implant placement procedures for each group.

Animals were randomly allocated into three experimental groups as follows:

- SHAM-SS: Animals underwent a simulated bilateral ovariectomy (sham surgery) at week 18 and received daily oral gavage of 0.5 mL of 0.9% saline solution starting from week 2.
- OVX-SS: Animals underwent bilateral ovariectomy at week 18 and received daily oral gavage of 0.5 mL of 0.9% saline solution starting from week 2.
- OVX-TAM: Animals underwent bilateral ovariectomy at week 18 and received daily oral gavage of tamoxifen citrate (15 mg/kg) [31] starting from week 2.

### 2.3. Animals

Ninety-six three-month-old rats (*Rattus norvegicus, albinus*, Wistar) weighing 300 ± 30 g were used and housed in plastic cages (three per group) under 12-hour light/dark cycles at 22 ± 2 °C, 55 ± 5% humidity, and 20 air changes/hour. They received feed and water ad libitum and had their cages changed weekly [32].

#### Sample Size Calculation and Randomization

The sample size was determined based on prior research [24,33,34] to achieve a statistical power of 0.8 and an alpha level of 0.05, considering a potential standard deviation of 12% and a minimal relevant difference of 10% between groups and time points. A total of twelve animals per time point was deemed sufficient to detect significant differences in bone-to-implant contact (BIC) and bone ingrowth (BIN). The study was designed as randomized, single-blind, and controlled. Randomization with a 1:1 allocation ratio was conducted using the Minitab^®^ software version 21.3.0 (Minitab Inc., State College, PA, USA).

### 2.4. Experimental Protocol

#### 2.4.1. Ovariectomy

Eighteen weeks before the beginning of the experiment, a bilateral ovariectomy was performed in the OVX groups following the protocol of Fiorin et al. (2022) [24]. In the SHAM group, the ovaries were lifted and returned to position. For all surgical procedures, rats were anaesthetized with ketamine (70 mg/kg body weight) and xylazine (6 mg/kg body weight) via intramuscular injection. Each animal received post-surgical intramuscular injections of 24,000 IU of penicillin G-benzathine (Pentabiotico Veterinario Pequeno Porte, Fort Dodge Saúde de Animal Ltda. Campinas, São Paulo, Brazil).

#### 2.4.2. Estrous Cycle

All rats underwent cytological examination to determine the cycle phase on Day 0. The vaginal cells were flushed by introducing the saline and drawing it with a modified pipette inserted at the entrance of the vaginal canal. The fluid was dropped in a slide and immediately analyzed under light microscopy at 400× magnification. The phase of the cycle was determined according to the presence of epithelial cells, cornified cells, and leukocytes in the cytological examination [35]. This step is important to ensure the success of the ovariectomy procedure.

#### 2.4.3. Systemic Treatments

The administration of either 0.5 mL of 0.9% saline solution or TAM was performed daily via oral gavage at a dosage of 15 mg/kg, two weeks after the estrous cycle verification. Systemic treatments were initiated four weeks prior to implant placement to evaluate their effects on early osseointegration. The dosage was determined based on previously established studies, employing a proportional adaptation method that considered body surface area for precise calculation [24,31].

#### 2.4.4. Implant Placement

Surgical procedures were performed under sedation and general anesthesia via intramuscular injection of xylazine hydrochloride (6 mg/kg) and ketamine hydrochloride (70 mg/kg). Following the protocol described by Fiorin et al. [24], for both tibiae, the implant sites (one per tibiae) were prepared perpendicularly to the long axis of the proximal metaphysis using a Ø2 mm implant drill under constant irrigation with saline solution, driven by a surgical motor (Ômega, Dentscler, Ribeirão Preto, São Paulo, Brazil) at 980 rpm. Each site received a conical titanium implant (4.0 × 2.2 mm) (DSP Biomedical^®^, Campo Largo, Paraná, Brazil) with the surface treated by sandblasting and dual acid etching, installed at the bone level. The soft tissues were sutured in two layers: internally with resorbable Vycril (horizontal mattress) and externally with non-resorbable simple interrupted sutures. In the post-operatory phase, the animals received a single dose of penicillin–streptomycin (0.1 mL/kg). For analgesia, morphine (2.5 mg/kg, intramuscular) was administered every 24 h for three days [24].

#### 2.4.5. Euthanasia and Sample Processing

At 7 and 30 days following implant placement, euthanasia was performed in twelve animals per experimental group via the intraperitoneal administration of sodium thiopental (Cristália Ltda., Itapira, São Paulo, Brazil) at a dose of 150 mg/kg. This procedure yielded 24 tibial specimens per group at each time point. All samples were fixed in 4% buffered formaldehyde for 48 h and subsequently processed either through undecalcified ground sectioning or by demineralization followed by paraffin embedding, according to the intended histological analysis.

#### 2.4.6. Ground-Section Processing

Ground sections were prepared according to a previously established protocol. [24,36]. Twelve tibias from each group and period underwent a gradual dehydration process using alcohol solutions containing fuchsine, followed by acetone washes and embedding in high-viscosity crystal resin (Arazyn 1.0#00, Redelease, São Paulo, São Paulo, Brazil). The samples were then polished using fine-grit abrasive paper (CarbiMet 2; Buehler, Lake Bluff, IL, USA) (#100, #400, #600, and #1200) until reaching 50 μm thickness.

#### 2.4.7. Demineralized Specimen Processing

Twelve tibias from each group and period underwent demineralization using a 10% ethylenediaminetetraacetic acid (EDTA) buffer. Samples were first embedded in paraffin with the implant in situ, ensuring the preservation of the bone structure adjacent to the implant’s thread and in order to avoid fractures. Afterwards, the implants were carefully removed using a hexagonal screwdriver and then re-embedded in paraffin. Semi-serial longitudinal sections, each 4 μm thick, were prepared along the area previously occupied by the implant. From each specimen, six evenly spaced sections from the central implant site were stained with hematoxylin and eosin (H&E) for histological and histometric evaluation of bone ingrowth and stained with Picrossirius Red for qualitative analysis of the maturation of the collagen fibers.

### 2.5. Data Analysis

Histological analyses were conducted by previously calibrated and blinded examiners, ensuring the absence of bias regarding group allocation. Two specific regions of interest (ROIs) [24,36] were delineated by a board-certified histologist (EE), a specialist in osteobiology, based on the spatial distribution and biological relevance of tissue responses—whether in pre-existing or newly formed peri-implant structures—triggered by implant placement. A representative illustration depicting the selected ROIs is provided (Figure 2).

ROI 1 was defined as the peri-implant compartment located between the implant threads, encompassing the area from the thread valley and extending 0.4 mm outward (equivalent to the depth of one thread). This region was selected for qualitative histological evaluation.

ROI 2 included the apical portion of the implant and comprised the second thread within cortical bone, as well as the first and second threads located in the bone marrow compartment. This region was designated for histometric analyses, specifically for the assessment of bone-to-implant contact (BIC) and bone ingrowth (BIN). ROI 2 was established bilaterally in relation to the original position of the implant.

#### 2.5.1. Histological Analysis

A histological description of the cellular and tissue reactions was performed under light microscopy (AxioLab^®^; Carl Zeiss, Gottingen, Germany), evaluating the following parameters: (1) pattern of cellularity and structure of peri-implant bone tissue; (2) pattern of cellularity and structure of peri-implant connective tissue; and (3) peri-implant inflammatory pattern.

#### 2.5.2. Histometric Evaluation of Bone-To-Implant Contact (BIC)

In the defined ROI 2, the extent of direct bone-to-implant contact (BIC) was quantified using the ImageJ software version 1.54d. The analysis consisted of measuring the linear interface where bone tissue was in direct contact with the implant surface. This value was then expressed as a percentage relative to the total perimeter of the implant threads within the region of interest [24,36].

#### 2.5.3. Analysis of the Bone Ingrowth Percentage

The percentage of bone ingrowth (BIN) within ROI 2 was determined using the ImageJ software. This analysis involved calculating the area occupied by bone tissue within the confines of the implant threads and expressing it as a percentage of the total inter-thread area [24,37].

#### 2.5.4. Polarization Technique with Picrosirius Red

The Picrosirius Red (PSR) polarization technique was employed to assess collagen fiber maturation under polarized light microscopy. Serial tissue sections were examined using polarized light to evaluate the organization, maturation, and density of collagen fi-ber bundles, based on the birefringence intensity of the collagen matrix. Image analysis was performed using the LAS software version 5.2.2 (400× magnification, Leica LAS, Leica Microsystems, Wetzlar, Germany), which allows for the precise definition of the color spectra corresponding to each type of collagen fiber. Following color selection, the software automatically calculated the percentage of fibers within the designated area. Greenish-yellow fibers were classified as immature, disorganized, and thin, whereas yellow-reddish fibers were considered mature, well-organized, and thick.

### 2.6. Primary and Secondary Outcomes

The primary outcome was defined as the new-formed bone in ROI 1, hence BIC (mm) and BIN (mm^2^). The secondary outcome was to define cellular events in vitro and in vivo, and to describe the structure of surrounding peri-implant tissues by histological and immunohistochemical analyses.

### 2.7. Statistical Analysis

Statistical analysis of the in vitro and in vivo data was conducted using BioEstat version 5.0 (Mamirua Institute, Manaus, Amazonas, Brazil). The Shapiro–Wilk test was applied to assess the normality of the data. For variables meeting normal distribution, a two-way ANOVA was performed to evaluate the effects of time and treatment, followed by Bonferroni post hoc testing. Statistical significance was set at *p* ≤ 0.05.

The experimental procedures were reviewed and approved by the Animal Ethics Committee (#520-2017) of Araçatuba Dental School, São Paulo State University, ensuring compliance with the National Council for Animal Experimentation Control (CONCEA). This research was conducted in accordance with the ARRIVE Guidelines.

## 3. Results

### 3.1. Tamoxifen Reduces SAOS-2 Viability and Collagen Deposition and Improves Mineralization Rate

**Figure 3:**
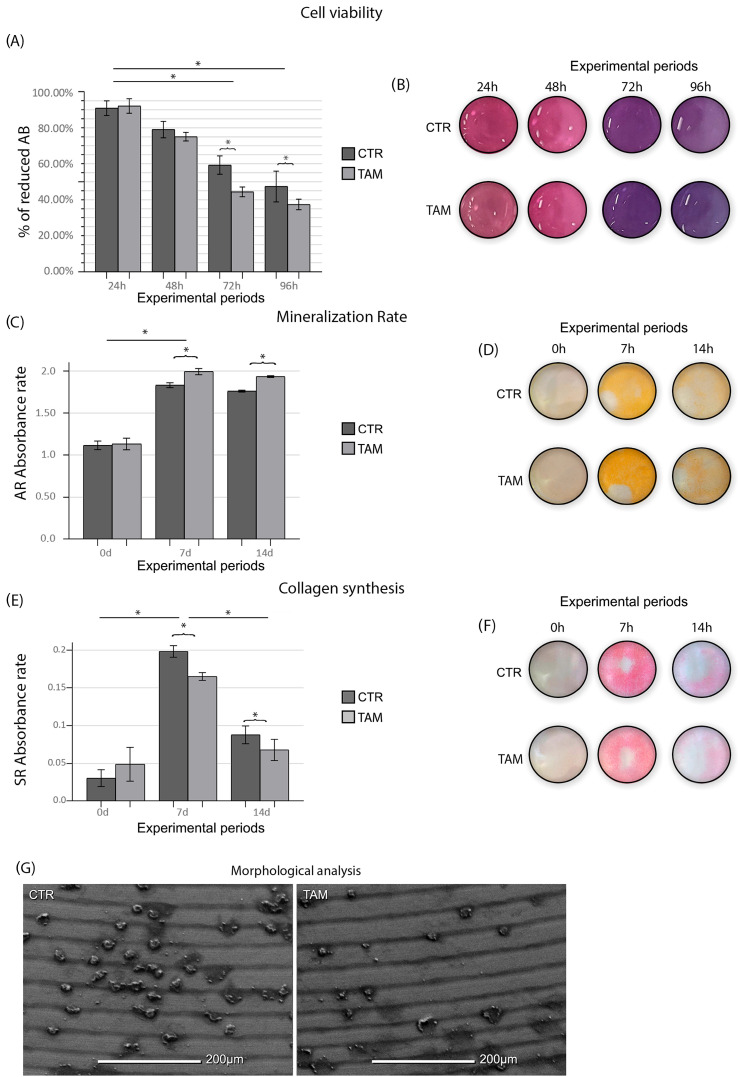
(**A**) Means and standard deviations (Ms ± SDs) of percentage of reduced Alamar Blue in each group and in each time point and (**B**) Alamar Blue (AB) supernatant depiction in the 96-well plate. (**C**) Means and standard deviations (Ms ± SDs) of the Alizarin Red (AR) absorbance rate of the cells in each group and (**D**) mineralized matrix deposition depicted at the bottom of the 6-well plate. (**E**) Means and standard deviations (Ms ± SDs) of the Sirius Red absorbance rate of the cells in each group and (**F**) collagen deposition depicted at the bottom of the 6-well plate. Statistical tests: ANOVA and Tukey. Symbols: * Statistically significant difference (*p* ≤ 0.05). (**G**) SEM photomicrographs showing the morphology of the SAOS-2-line cells in the CTR and TAM groups at 96 h. Original magnification: 500×. Scale bar: 200 μm.

As evidenced by the ANOVA test (*p* ≤ 0.05), the enrichment of TAM in the culture medium caused a lower SAOS-2 viability at 72 h and 96 h when compared to the CTR group (*p* ≤ 0.05) (Figure 3). The TAM group (1.13 ± 0.06; 1.99 ± 0.03; 1.93 ± 0.01) presented a higher mineralization rate at 7 d and 14 d when compared to the CTR group (1.11 ± 0.05; 1.83 ± 0.02; 1.76 ± 0.01) (*p* ≤ 0.05) (Figure 3). The TAM group (0.049 ± 0.022; 0.165 ± 0.005; 0.068 ± 0.014) presented lower collagen synthesis at 7 d and 14 d when compared to the CTR group (0.031 ± 0.011; 0.198 ± 0.008; 0.088 ± 0.012) (*p* ≤ 0.05) (Figure 3).

SEM observation showed in both groups, TAM and CTR, a titanium surface characterized by scattered cell groups. SAOS-2 showed a round and withered appearance compared to their physiological morphology. No evident morphological alterations were observed among the viable cells across the experimental groups. However, in the groups treated with tamoxifen, a notable increase was detected in the number of cells exhibiting circular membrane protrusions, a feature suggestive of early detachment from the implant surface (Figure 3).

### 3.2. Tamoxifen Increases Bone/Implant Contact and Bone Ingrowth and Reduces Foci of Inflammation

**Figure 4:**
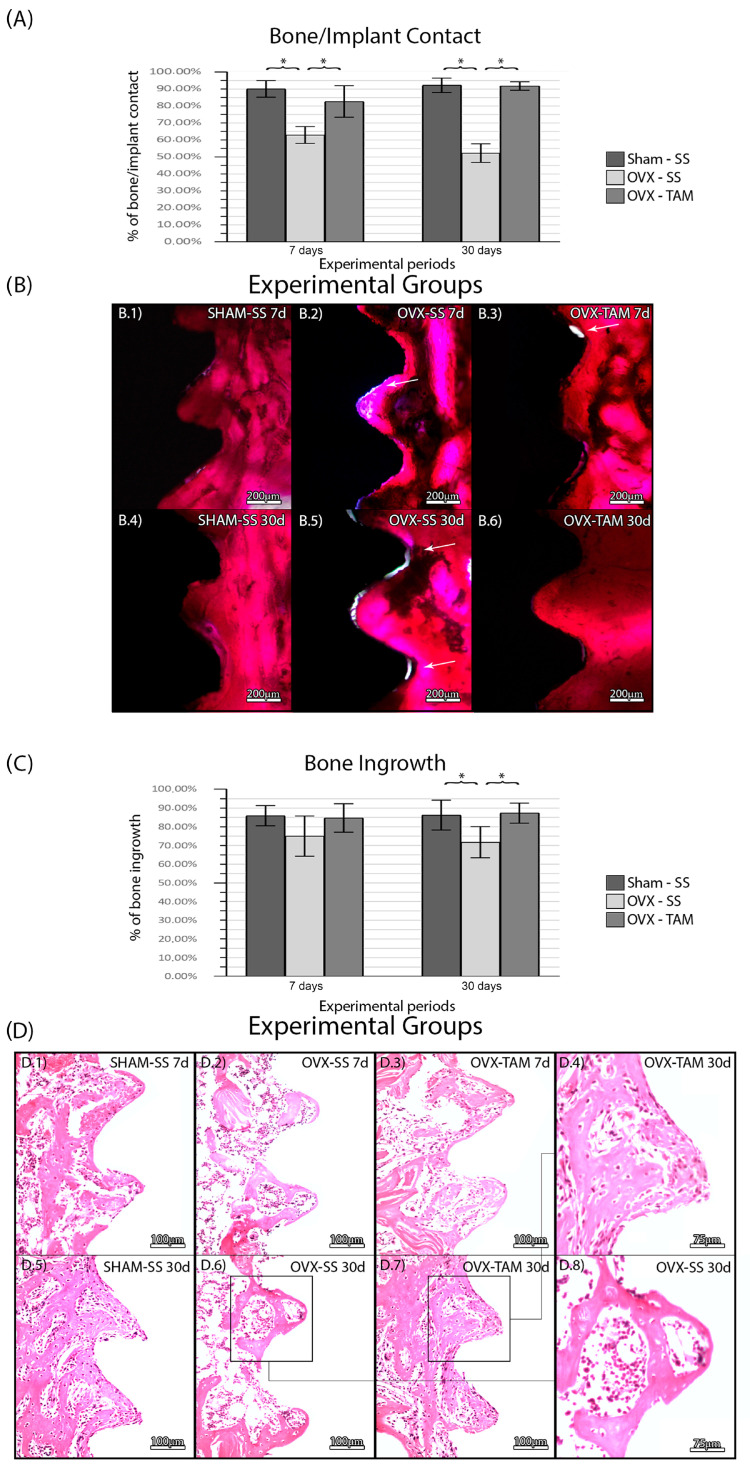
(**A**) Bone/implant contact for each experimental group. Means and standard deviations (Ms ± SDs) of BIC for each group and period. (**B**) Photomicrographs of BIC in the SHAM-SS (**B.1**,**B.4**), OVX-SS (**B.2**,**B.5**), and OVX-TAM (**B.3**,**B.6**) groups. White arrows: Areas without bone/implant contact. Staining: Basic fuchsin. Scale bar: 200 μm. Bone percentage area for each experimental group. Means and standard deviations (Ms ± SDs) of the BIN for each group and period (**C**). Photomicrographs showing the histological features of the peri-implant tissues for each group. (**D**) Photomicrographs of the BIN in the SHAM-SS (**D.1**,**D.5**), OVX-SS (**D.2**,**D.6**,**D.8**), and OVX-TAM (**D.3**,**D.4**,**D.7**) groups. Staining: Hematoxylin and eosin. Scale bars: 100 μm and 75 μm. Statistical tests: ANOVA and Bonferroni. Symbols: * Statistically significant difference (*p* ≤ 0.05).

No complications were observed in the animals throughout the entire duration of the experimental protocol. There was no statistical difference between SHAM-SS (90.17%± 4.85%; 92.29%± 4.29%) and OVX-TAM (82.74% ± 9.31%; 91.84% ± 2.49%) in all periods. The OVS-SS group (63.00% ± 4.93%; 52.32% ± 5.48%) presented a lower bone/implant percentage in all periods compared to the other groups (*p* ≤ 0.05) (Figure 4). There was no statistical difference between SHAM-SS (85.96 ± 5.40%; 86.31 ± 8.01%) and OVX-TAM (84.76 ± 7.62%; 87.35% ± 5.38%) in all periods. The OVS-SS group (75.06 ± 10.73%; 71.82 ± 8.34%) presented a lower bone ingrowth percentage in all periods compared to all groups (*p* ≤ 0.05) (Figure 4).

In the SHAM-SS and in the OVX-TAM groups, bone tissue presented a physiological aspect at D7 and D30 with a predominance of vital bone tissue observed in the region of interest. In both cancellous and compact bone, few active osteoclast-like cells were observed. In some regions, particularly at D30, a small amount of connective tissue was observed near the bone tissue, with only a few areas of inflammatory foci. Only a minimal amount of non-vital bone was observed. In the OVX-SS group, the overall tissue seemed pathological, with a large area of connective tissue rich in hematopoietic tissue. The larger area of connective tissue and foci of inflammation, compared to the other two groups, reduced the total area of bone tissue (Figure 3).

**Figure 5:**
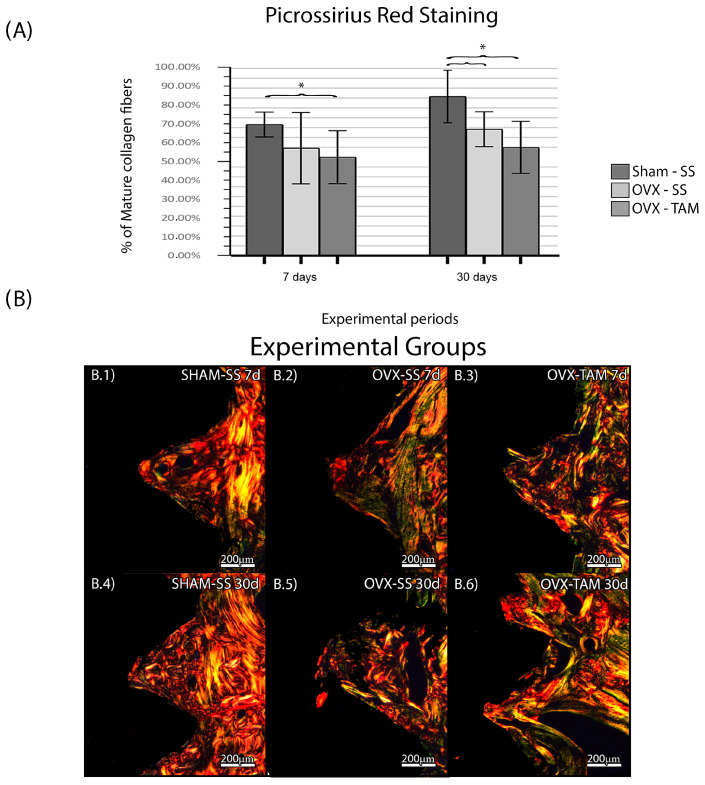
Percentage of mature collagen fibers. Means and standard deviations (Ms ± SDs) of PSR for each group and period (**A**). (**B**) Photomicrographs of the PSR in the SHAM-SS (**B.1**,**B.4**), OVX-SS (**B.2**,**B.5**), and OVX-TAM (**B.3**,**B.6**) groups. Staining: Picrossirius Red. Scale bar: 200 μm. Statistical tests: ANOVA and Bonferroni. Symbols: * Statistically significant difference (*p* ≤ 0.05).

The SHAM-SS group presented a higher percentage of mature collagen fibers in the period of 30 days (66.6 ± 6.29%; 80.8% ± 13.36%) when comparing to OVX-SS (54.6 ± 18.14%; 64.2 ± 8.82%) and OVX-TAM (51.10 ± 13.21%; 55.40 ± 13.13%), which presented the lowest percentage of mature collagen fibers (Figure 5).

## 4. Discussion

The present study examines the effects of tamoxifen (TAM) on osseointegration, especially in postmenopausal contexts where breast cancer and subsequent ER+ tumors are common. In approximately 80% of breast cancer cases diagnosed in postmenopausal women, ER+ tumors proliferate in response to estrogen [38,39], making estrogen-modulating therapies like TAM a primary treatment option.

SAOS-2 cells were selected to study the secondary effects of tamoxifen in the context of titanium implant installation in bone. These cells express both subtypes of estrogen receptors, α and β [40,41], and exhibit proliferation and osteogenic differentiation rates comparable to primary osteoblasts [42,43], making them a suitable model when compared to other osteoblastic cell lines.

The tamoxifen dosage in vitro was chosen based on the study by Darakhshan et al. (2015) [27], which demonstrated that a concentration of 2 µM maintained an acceptable cell viability percentage while exhibiting minimal toxicity across multiple breast cancer cell lines. Higher concentrations, such as 5 µM, were associated with cessation of cell replication, while 10 µM and 20 µM presented significantly higher toxicity, as observed by Majumdar et al. (2001) [44].

In early periods, tamoxifen-treated groups exhibited higher cell growth compared to the DMEM group. This effect may be attributed to tamoxifen’s protective role in osteoblastic cells [10], mediated through both estrogen receptors ERα and ERβ [9,45], as previously described by Kallio et al. (2008) [9]. However, a decrease in viability was observed after 96 h, confirmed by SEM, which revealed a substantial number of cells with a rounded morphology characteristic of apoptosis. These findings suggest that, while tamoxifen exerts an early stimulatory effect on osteoblastic activity, prolonged exposure may trigger apoptotic and anti-proliferative pathways, potentially through estrogen receptor-independent mechanisms [46,47,48], as observed also by Pike (2015) [49] on other in vitro models. Furthermore, titanium (Ti) itself was shown to negatively affect the viability of SAOS-2 cells [50,51,52], which may have contributed to the observed late cytotoxic effects.

Collagen deposition, a critical step for matrix mineralization, initially increases across all groups by Day 7 but decreases over time as the matrix mineralizes. Tamoxifen negatively influenced matrix organization [52] and remodeling, resulting in reduced collagen synthesis rates in the tamoxifen-treated groups. In vivo, collagen deposition plays a crucial role in intramembranous ossification and bone tissue mineralization [53]. Despite reduced viability and collagen synthesis, tamoxifen enhanced the mineralization rate of SAOS-2 cells in vitro independent of the activation of the ER receptor, a phenomenon reported as early as 1995 [23].

Osteoblast mineralization occurring in the absence of collagen deposition is an uncommon phenomenon, given that collagen, particularly type I collagen, typically serves as the structural scaffold for mineral deposition in bone. Nonetheless, several experimental and biological contexts have demonstrated that mineralization and collagen synthesis can be at least partially uncoupled. For instance, the experimental inhibition of collagen production using agents such as ethyl-3,4-dihydroxybenzoate (DHB) has shown that, even when collagen synthesis is suppressed, mineralization markers such as alkaline phosphatase and osteocalcin may still be upregulated, and mineral nodules can form, albeit abnormally or with reduced efficiency (Zhu et al., 2023) [54]. Moreover, certain non-collagenous proteins, including osteonectin [54] (SPARC) and bone sialoprotein (BSP) [55], are known to promote mineral deposition independently of the collagen matrix, due to their high affinity for hydroxyapatite and regulatory roles in mineralization. The matrix vesicle pathway [56], active during early developmental stages or in vitro conditions, can also initiate mineralization prior to the establishment of a mature collagen scaffold. Additionally, genetic or signaling pathway disruptions, such as knockouts of *EphrinB2* or *DDR2*, have been shown to decouple collagen deposition from mineralization, resulting in mineralized but structurally deficient bone tissue (Blank & Sims, 2019) [57]. The present findings may represent an additional example of this phenomenon, in line with these reported cases. Further investigation will be required to elucidate the specific molecular mechanisms involved, which will be addressed in a dedicated, targeted follow-up study.

Concerning in vivo experiments, despite limitations such as the absence of bacterial challenges and occlusal forces, the pattern of bone loss in rodents closely mimics human bone behavior [58,59,60]. In this study, ovariectomized (OVX) rats were chosen as they are widely recognized in the literature as an optimal model for evaluating postmenopausal bone metabolism [61,62,63,64]. Since tamoxifen (TAM) is primarily administered as adjunctive therapy in postmenopausal women, this model reliably simulates the clinical scenario of implant therapy in postmenopausal patients and helps elucidate the isolated effects of TAM on the osseointegration process. In vivo, the TAM dosage was selected aiming to lower the incidence of TAM resistance [31] and important drug effect, as observed in previous studies [24]. Higher concentrations of TAM cause liver and kidney toxicity [65] and are lethal for female rats [66].

Bone–implant contact (BIC) is the primary outcome for evaluating the success of osseointegration [67,68], as it reflects the intimate contact between bone tissue and the titanium implant threads. Systemic factors significantly influence this contact. In this context, tamoxifen acts as an estrogen agonist in bone tissue. This effect was evident in the TAM-treated groups (OVX-TAM), which exhibited higher BIC compared to the control group (OVX-SS), although without significant alterations in histological structure at 7 and 30 days after implant placement, with similar characteristics to the negative control group (SHAM-SS).

Similarly, previous studies have reported that TAM positively impacts bone mass maintenance and mineral density in postmenopausal women. In addition, TAM presented a positive effect in the bone remodeling around osseointegrated implants [23]. Furthermore, not all systemic conditions are reflected in the bone/implant contact. Bone ingrowth is an important factor for the long-term success of the dental implants and is characterized by bone that grows between the implant threads [69]. In line with the literature, which shows TAM has an estrogen agonist effect [70,71], we observed a higher BIN in the TAM groups when compared to the control groups.

Although the groups in which TAM was administered presented higher BIC and BIN, they also presented lower percentage of mature collagen fibers. Studies have demonstrated that mineral deposition can occur in the presence of immature collagen fibrils, especially in an environment where the resorption is controlled. Tamoxifen reportedly suppresses osteoclastic activity and increases osteoblastic differentiation, thereby promoting mineralization independently of collagen fiber maturation. However, paradoxically, we can hypothesize that the increase in BIC and BIN is a result from favorable conditions for mineral deposition than from increased matrix organization.

As a limitation of our study, the implants were not exposed to bacterial contamination from the oral cavity or subjected to occlusal loading. Additionally, the experimental design does not allow for a proper evaluation of the long-term secondary effects of tamoxifen exposure in premenopausal animals. In premenopausal women, studies have shown that tamoxifen has limited effectiveness in treating breast cancer [72] and may increase the risk of bone fractures [73]. This phenomenon remains unclear in the literature but may be related to the interaction between tamoxifen and estrogen receptors. In this population, the presence of normal physiological levels of endogenous sex hormones, combined with the modulatory activity of tamoxifen, which can act as an estrogen agonist or antagonist depending on the tissue and hormonal context, may result in additive or conflicting signaling [74]. This dual stimulation could alter receptor sensitivity and help explain the contrasting effects of tamoxifen observed in estrogen-sensitive tissues, including bone.

Based on the findings of these experiments, the therapy with TAM in vitro slightly reduced the viability of SAOS-2 cells while increasing the mineralization rate. In vivo, the therapy positively influenced BIC and BIN during the osseointegration phase. These results suggest that titanium implants can be predictably and safely installed in patients undergoing tamoxifen therapy. Nonetheless, further studies are necessary to evaluate these outcomes under occlusal loading and mechanical stress and under bacterial challenges.

## 5. Conclusions

Tamoxifen showed to be stimulating for the mineralization rate and to positively influence BIC and BIN in ovariectomized animals, suggesting that tamoxifen administration in postmenopausal women does not harm implant osseointegration.

## Data Availability

The original contributions presented in this study are included in the article/Supplementary Materials. Further inquiries can be directed to the corresponding author.

## Supplementary Materials

The following supporting information can be downloaded at: https://www.mdpi.com/article/10.3390/dj13080351/s1, Table S1: Cell Viability, Table S2: Mineralization Rate, Table S3: Collagen Synthesis, Table S4: Bic, Table S5: Bin, Table S6: Picrossirius.
