# Supplementary material for: Behavior of Osteoblastic Lineage Cells When in the Presence of Tamoxifen: In Vitro and In Vivo Studies on Osseointegration
Source: Dent J (Basel). 2025 Aug 1;13(8):351. doi: 10.3390/dj13080351 (PMC12384849; doi:10.3390/dj13080351)
Supplement: Supplementary file 1 [file dentistry-13-00351-s001.zip › dentistry-3529495-supplementary.pdf]

Cell Viability											
24h			48h			72h			96h		
Sample	CTR	TAM	Sample	CTR	TAM	Sample	CTR	TAM	Sample	CTR	TAM
disk 1	82,90	87,60	disk 1	82,40	77,10	disk 1	59,98	47,64	disk 1	53,08	37,34
	88,81	95,80		79,90	74,30		55,96	46,97		49,34	41,23
	98,74	94,01		79,90	75,70		61,26	40,36		49,33	38,16
disk 2	87,95	89,87	disk 2	67,50	75,80	disk 2	54,09	44,46	disk 2	63,76	42,90
	87,23	89,05		82,76	71,20		58,83	47,22		57,58	38,51
	91,57	95,70		74,70	74,50		50,95	43,40		52,57	35,07
disk 3	90,15	87,50	disk 3	81,20	72,00	disk 3	59,77	44,33	disk 3	49,00	36,38
	95,44	93,30		77,80	80,20		54,98	38,51		35,58	35,23
	88,96	99,12		78,70	73,90		71,36	43,93		37,01	35,41
disk 4	89,82	90,90	disk 4	81,80	77,60	disk 4	61,91	45,05	disk 4	45,46	41,96
	91,35	97,40		83,90	72,80		59,79	46,43		41,94	34,50
	95,54	98,40		73,40	71,80		58,11	46,97		59,67	34,14
disk 5	92,71	92,00	disk 5	79,80	75,80	disk 5	59,56	39,93	disk 5	50,10	39,94
	91,69	89,04		81,80	76,40		51,61	45,30		46,01	33,30
	88,31	89,00		83,40	77,60		57,23	42,16		51,84	41,16
disk 6	97,50	95,30	disk 6	76,40	73,90	disk 6	59,33	44,11	disk 6	38,63	37,01
	85,76	87,60		73,50	75,40		65,8	44,85		34,45	37,46
	92,29	87,10		84,90	77,20		66,66	47,53		38,12	35,17
Sum	**90,93**	**92,15**	Sum	**79,10**	**75,18**	Sum	**59,29**	**44,40**	Sum	**47,42**	**37,49**
SD	4,08	4,06	SD	4,52	2,36	SD	5,12	2,70	SD	8,51	2,91

Mineralization Rate										
0d				7d				14d		
Sample	CTR	TAM		Sample	CTR	TAM		Sample	CTR	TAM
disk 1	0,960	1,140		disk 1	1,850	1,990		disk 1	1,770	1,920
	1,130	1,120			1,850	1,930			1,770	1,920
	1,200	1,190			1,840	1,990			1,760	1,930
disk 2	1,130	1,110		disk 2	1,780	2,020		disk 2	1,760	1,940
	1,140	1,120			1,790	2,030			1,770	1,930
	1,120	1,230			1,840	1,960			1,770	1,940
disk 3	1,110	1,100		disk 3	1,810	2,000		disk 3	1,760	1,930
	1,130	1,140			1,820	1,990			1,750	1,940
	1,150	1,060			1,820	2,040			1,740	1,930
disk 4	1,110	1,190		disk 4	1,900	2,010		disk 4	1,750	1,920
	1,200	1,210			1,810	1,990			1,770	1,940
	1,080	1,000			1,830	2,030			1,780	1,960
disk 5	1,070	0,990		disk 5	1,820	1,930		disk 5	1,760	1,930
	1,120	1,110			1,870	2,010			1,750	1,940
	1,100	1,160			1,820	1,970			1,750	1,940
disk 6	1,100	1,090		disk 6	1,840	2,050		disk 6	1,750	1,940
	1,130	1,160			1,810	1,940			1,770	1,920
	1,080	1,230			1,850	1,980			1,760	1,930
Sum	**1,114**	**1,131**		Sum	**1,831**	**1,992**		Sum	**1,761**	**1,933**
Standart De	0,052	0,069		Standart De	0,028	0,036		Standart De	0,011	0,010

Collagen sinthesis										
0d				7d				14d		
Raw Data	CTR	TAM		Raw Data	CTR	TAM		Raw Data	CTR	TAM
disk 1	0,010	0,060		disk 1	0,190	0,160		disk 1	0,080	0,080
	0,020	0,050			0,190	0,160			0,090	0,080
	0,050	0,030			0,210	0,170			0,100	0,080
disk 2	0,040	0,030		disk 2	0,200	0,160		disk 2	0,080	0,080
	0,040	0,070			0,200	0,160			0,080	0,060
	0,030	0,010			0,200	0,170			0,110	0,050
disk 3	0,040	0,040		disk 3	0,190	0,170		disk 3	0,080	0,070
	0,020	0,010			0,190	0,160			0,100	0,090
	0,040	0,070			0,190	0,170			0,090	0,060
disk 4	0,020	0,090		disk 4	0,210	0,160		disk 4	0,080	0,080
	0,020	0,030			0,200	0,170			0,090	0,050
	0,030	0,060			0,200	0,170			0,080	0,060
disk 5	0,040	0,060		disk 5	0,190	0,170		disk 5	0,090	0,060
	0,020	0,070			0,210	0,160			0,090	0,070
	0,040	0,070			0,210	0,170			0,090	0,090
disk 6	0,020	0,030		disk 6	0,200	0,160		disk 6	0,070	0,050
	0,030	0,040			0,200	0,170			0,110	0,050
	0,040	0,060			0,190	0,160			0,070	0,060
Sum	**0,031**	**0,049**		Sum	**0,198**	**0,165**		Sum	**0,088**	**0,068**
SD	0,011	0,022		SD	0,008	0,005		SD	0,012	0,014

BIC						
	1	2	3	4	5	6
Sample	**Sham SS7D**	**Sham SS 30**	**OVX Tam 7**	**OVX Tam 30**	**OVX SS 7**	**OVX SS 30**
R1	93,89%	98,08%	70,97%	90,10%	63,02%	51,46%
R2	98,52%	98,08%	93,87%	90,45%	65,29%	60,14%
R3	90,06%	93,08%	92,10%	94,46%	51,41%	44,77%
R4	83,82%	88,22%	90,97%	90,60%	62,33%	51,46%
R5	91,00%	94,93%	70,97%	90,45%	72,20%	60,14%
R6	91,46%	87,07%	90,97%	94,46%	63,02%	44,77%
R7	83,82%	87,07%	70,97%	90,60%	62,33%	52,00%
R8	89,30%	89,70%	87,70%	94,50%	63,45%	46,95%
R9	83,20%	90,21%	89,12%	89,31%	68,65%	48,54%
R10	89,82%	88,56%	81,30%	95,65%	62,81%	54,99%
R11	90,43%	96,63%	82,36%	93,44%	60,45%	54,15%
R12	96,69%	95,85%	71,59%	88,11%	61,09%	58,53%
Sum	**90,17%**	**92,29%**	**82,74%**	**91,84%**	**63,00%**	**52,32%**
SD	**4,85%**	**4,29%**	**9,31%**	**2,49%**	**4,93%**	**5,48%**

BIN						
	1	2	3	4	5	6
Sample	**Sham SS7D**	**Sham SS 30**	**OVX Tam 7**	**OVX Tam 30**	**OVX SS 7**	**OVX SS 30**
R1	87,12	97,38	82,01	91,33	86,33	72,55
R2	82,98	94,20	94,33	82,21	73,21	82,09
R3	91,86	80,82	80,48	93,81	70,74	78,47
R4	83,12	94,27	68,85	93,78	83,37	59,89
R5	87,41	83,30	94,30	80,71	53,89	68,21
R6	88,58	89,52	81,12	94,52	81,43	71,74
R7	79,43	76,96	82,04	85,84	60,19	83,56
R8	86,78	91,40	77,11	83,12	71,64	60,14
R9	94,95	70,38	89,27	79,36	80,38	64,98
R10	86,67	90,78	90,22	86,89	79,67	64,07
R11	74,59	82,08	91,61	85,88	69,2	77,3
R12	88,02	84,62	85,82	90,75	90,65	78,82
Sum	**85,96**	**86,31**	**84,76**	**87,35**	**75,06**	**71,82**
SD	**5,40**	**8,01**	**7,62**	**5,38**	**10,73**	**8,34**

Picrossirius

Samples	Sham - SS 7	OVX-SS 7	OVX-TAM 7	Sham - SS 30	OVX-SS 30	OVX-TAM 30
R1	55,00%	47,00%	33,00%	62,00%	78,00%	31,00%
R2	60,00%	25,00%	71,00%	60,00%	51,00%	77,00%
R3	62,00%	39,00%	38,00%	75,00%	71,00%	46,00%
R4	70,00%	87,00%	58,00%	85,00%	76,00%	69,00%
R5	68,00%	72,00%	35,00%	90,00%	55,00%	45,00%
R6	75,00%	46,00%	49,00%	98,00%	58,00%	53,00%
R7	73,00%	45,00%	45,00%	88,00%	60,00%	52,00%
R8	66,00%	70,00%	55,00%	95,00%	62,00%	57,00%
R9	72,00%	55,00%	62,00%	70,00%	65,00%	61,00%
R10	65,00%	60,00%	65,00%	85,00%	66,00%	63,00%
R11	60,63%	37,39%	38,57%	68,13%	55,84%	42,94%
R12	72,57%	71,81%	63,63%	93,47%	72,56%	67,86%
**SUM**	66,60%	54,60%	51,10%	80,80%	64,20%	55,40%
**SD**	6,24%	17,98%	13,09%	13,23%	8,74%	13,01%
